# Chemical Composition and Larvicidal Activity Against *Aedes aegypti* of the Leaf Essential Oils from *Croton blanchetianus*

**DOI:** 10.3390/molecules30051034

**Published:** 2025-02-24

**Authors:** Pedro Henrique Ribeiro Lopes, Nicaely Maria de Oliveira Pereira, Matheus Nunes da Rocha, Marcia Machado Marinho, Jesyka Macêdo Guedes, Tigressa Helena Soares Rodrigues, Jean Parcelli Costa Do Vale, Emmanuel Silva Marinho, Gilvandete Maria Pinheiro Santiago, Hélcio Silva dos Santos

**Affiliations:** 1 Postgraduate Program in Natural Sciences, Ceará State University, Fortaleza 60714-903, CE, Brazil; hlopes2906@gmail.com (P.H.R.L.); matheusndarocha@gmail.com (M.N.d.R.); marcia.marinho@uece.br (M.M.M.); jesyka.mg@gmail.com (J.M.G.); emmanuel.marinho@uece.br (E.S.M.); 2 Department of Organic and Inorganic Chemistry, Federal University of Ceará, Fortaleza 60020-181, CE, Brazil; nicaely99oliveira@gmail.com (N.M.d.O.P.); gil@ufc.br (G.M.P.S.); 3 Center for Exact Sciences and Technology, Vale do Acaraú University, Sobral 62040-370, CE, Brazil; thelenasr@yahoo.com.br (T.H.S.R.); jeanvale@hotmail.com (J.P.C.D.V.); 4 Department of Pharmacy, Federal University of Ceara, Fortaleza 60430-160, CE, Brazil

**Keywords:** *Croton blanchetianus*, essential oils, circadian rhythm, *Aedes aegypti*

## Abstract

The *Aedes aegypti* mosquito is the primary vector of dengue, a neglected disease and a serious public health problem in tropical countries. The control of this vector has been carried out using chemical insecticides, which impact human health. Thus, it is essential to develop natural larvicides that are less harmful to the environment. This study investigates the circadian cycle and larvicidal activity of essential oils from *Croton blanchetianus* against *Aedes aegypti*. The leaf oils were extracted by hydrodistillation and analyzed by GC–MS and GC–FID. The circadian study revealed variations in the chemical composition of oils extracted at different times of the day. The main constituents were *α-pinene*, *β-phellandrene*, *eucalyptol*, *β-caryophyllene*, *bicyclogermacrene*, and *spathulenol*. The larvicidal activity showed LC_50_ values at the following different collection times: 55.294 ± 3.209 μg/mL at 08:00 h; 95.485 ± 2.684 μg/mL at 12:00 h; and 64.883 ± 1.780 μg/mL at 17:00 h. Molecular docking simulations indicated that *α-pinene*, *β-phellandrene*, *eucalyptol*, and *β-caryophyllene* strongly interact with the active site of the sterol carrier protein, suggesting their role in larvicidal activity. These findings reinforce the potential of *C. blanchetianus* essential oils as an alternative for *Aedes aegypti* control. The predictive pharmacokinetic tests showed a PAMPA profile associated with high effective cellular permeability and microsomal stability, resulting from the metabolic stability of the derivatives (3) *eucalyptol* and (6) *spathulenol*, indicating that these compounds have the highest pharmacokinetic viability and low reactivity with respect to organ toxicity.

## 1. Introduction

Dengue is considered the most significant arbovirus disease, affecting more than half of the world’s population in endemic areas. A global estimate suggests that between 50 and 200 million dengue cases occur annually, resulting in approximately 20,000 deaths [1,2]. In Brazil, the situation is particularly alarming. By October 2024, the country had recorded approximately 6.5 million probable cases of dengue, according to the Arbovirus Monitoring Panel of the Ministry of Health [3]. This demonstrates an urgent need for improved vector control strategies. Over the past 30 years, the number of countries reporting dengue outbreaks has increased tenfold, with the disease now present in at least 100 tropical and subtropical nations, including Africa, Southeast Asia, the Western Pacific, the Americas, the Caribbean, and the Eastern Mediterranean [4].

The economic burden of dengue is substantial, with the global costs for medical treatment, surveillance, vector control, and lost productivity estimated at approximately $39 billion per year. In the Americas, the annual cost of illness is estimated to be between $1 and $4 billion [5].

Currently, vector control relies heavily on the use of chemical insecticides, particularly organophosphates and pyrethroids. However, the extensive use of these substances has led to serious issues, including insecticide resistance in *Aedes aegypti* and environmental pollution [6]. Resistance mechanisms involve target site insensitivity, metabolic detoxification, and behavioral adaptations, which reduce the effectiveness of traditional chemical control methods [7,8]. Given this challenge, there is an increasing need for alternative strategies such as the use of plant-derived compounds with larvicidal potential.

In recent years, the search for natural larvicidal compounds with low environmental toxicity has intensified, making plant-derived products a promising alternative for mosquito control. Essential oils (EOs) have emerged as particularly attractive candidates due to their biodegradability, availability, and potential larvicidal activity against *Aedes aegypti* [9].

**Figure 1:**
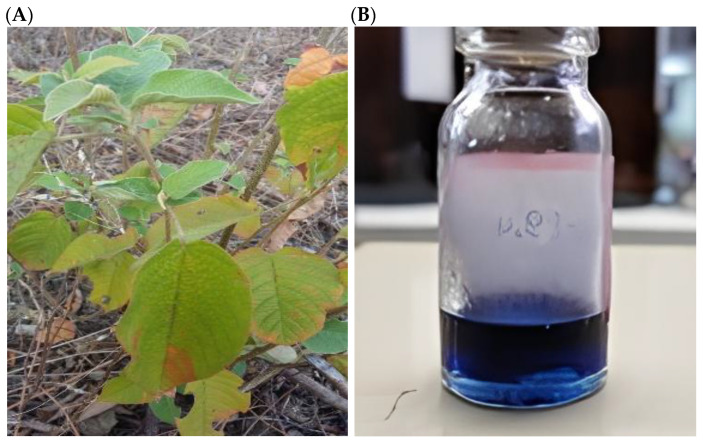
*Croton blanchetianus* (**A**), blue essential oil *C. blanchetianus* (**B**).

The genus *Croton* (Euphorbiaceae) (Figure 1) is the second largest in its family, comprising approximately 1300 species distributed throughout tropical and subtropical regions. Many *Croton* species have been traditionally used in folk medicine to treat various ailments [10]. Additionally, several studies have reported the phytochemical composition and biological activities of essential oils extracted from *Croton* species [11,12,13,14,15].

*C. blanchetianus* is an endemic species in northeastern Brazil, commonly found in the Caatinga biome, and is known as “velame” and “marmeleiro”. Although some research has been conducted on the phytochemical characterization of other *Croton* species, there is a lack of studies specifically addressing the chemical composition and biological properties of *C. blanchetianus* essential oil, particularly its larvicidal potential against *Aedes aegypti* [16].

Furthermore, the chemical composition of essential oils can vary depending on the circadian cycle, influencing their biological activity. Studies suggest that essential oil yield and composition fluctuate throughout the day due to variations in metabolic activity, light exposure, and environmental conditions. However, the impact of these variations on larvicidal efficacy remains largely unexplored [17].

Among the major constituents of *C. blanchetianus* essential oil, *α-pinene* and *β-caryophyllene* have been previously identified as effective against the larvae of mosquitoes transmitting malaria, dengue, and Japanese encephalitis. These compounds exhibit neurotoxic and metabolic-disrupting properties, making them promising candidates for vector control. However, their specific modes of action in *Aedes aegypti* remain to be further investigated [18].

In this context, the present work aimed to evaluate the influence of the circadian cycle and larvicidal activity against *Aedes aegypti* of essential oils from the leaves of *C. blanchetianus*.

## 2. Results

### 2.1. Chemical Composition

Essential oils extracted from the leaves of *C. blanchetianus* at 8 h, 12 h, and 17 h were analyzed by GC–MS and GC–FID. The chemical composition of the oils, including the retention index and the percentage relative to each constituent, are presented in Table 1. The major components were the monoterpenes *α-pinene*, *β-phellandrene*, *eucalyptol*, and the sesquiterpenes *β-caryophyllene*, *bicyclogermacrene*, and *spathulenol*.

A total of 23 constituents (89.56%) were identified in the essential oil extracted at 8 h, comprising 13 monoterpenes (41.49%) and 10 sesquiterpenes (48.07%). For the oil extracted at 12 h, 25 constituents (98.27%) were identified, with 16 monoterpenes (61.35%) and 9 sesquiterpenes (36.92%). At 17 h, 16 constituents (100.00%) were found, including 9 monoterpenes (48.00%) and 7 sesquiterpenes (52.00%).

### 2.2. Larvicidal Activity

The larvicidal activity of *C. blanchetianus* essential oils against *Aedes aegypti* was evaluated using Temephos^®^ (*O*,*O*′-(thiodi-p-phenylen)bis(*O*,*O*′dimethylthiophosphate)) as a positive control. Mortality percentages were calculated after 24 h. The larvicidal activity of these essential oils is presented in Table 2. The LC_50_ values were 55.29 ± 3.21 μg/mL (8 h); 95.48 ± 2.68 μg/mL (12 h); and 64.88 ± 1.78 μg/mL (17 h) (Table 2).

The larvicidal activity of essential oils of some species from the genus *Croton* has been previously reported on; for instance, essential oils from leaves, stalks and inflorescences of *C. zehntneri* and *C. jacobinensis* showed LC_50_ values of 56.2, 51.3, 57.5 and 79.3, 117.2, 65.8 μg/mL, respectively, and were tested at different concentrations against *Aedes aegypti* [19,20]. Essential oils from the aerial parts of *C. argyrophylloides*, and *C. nepetaefolius* showed LC_50_ values of 94.6, and 66.4 μg/mL, respectively [21], whereas essential oils from the leaves of *C. regelianus* growing at two different sites in Ceará State (Brazil) showed LC_50_ values of 24.22 and 66.74 μg/mL [22].

The mortality percentages were calculated after 24 h; the larvicidal effects of these essential oils are shown in Table 2. The essential oils obtained at 6, 12 and 17 h showed LC_50_ values of 55.294, 95.485, and 64.883 μg/mL, respectively. Therefore, the different activities of the essential oils can be attributed to variations in their chemical compositions. Essential oils from the leaves of *C. blanchetianus* demonstrated larvicidal activity against *Aedes aegypti*, which can be explained by a possible relationship between larvicidal activity and the presence of monoterpenes (*α-pinene*, sabinene, β-pinene, myrcene, α-terpinene, and γ-terpinene, terpinen-4-ol) and sesquiterpenes (*β-caryophyllene* and *Bicyclogermacrene*) which have been reported to be active against *Aedes aegypti* and can serve to increase the transmembrane absorption of lipophilic drugs, which can kill the larvae of *Aedes aegypti* and mediate synergistic effects [23,24,25].

### 2.3. Molecular Docking

**Figure 2:**
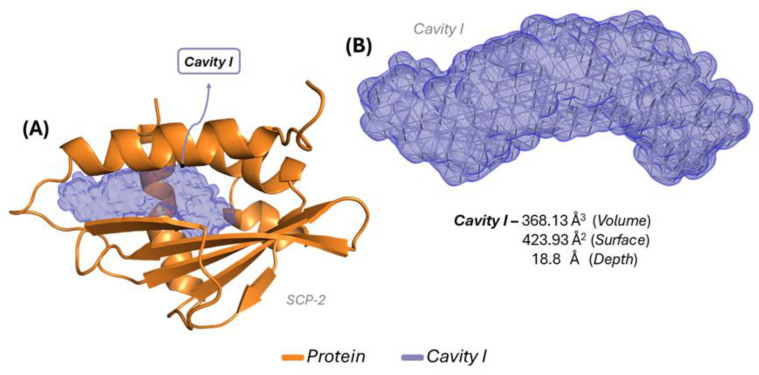
SCP-2 receptor interaction cavity: (**A**) representation of the cavity inserted into the receptor; and (**B**) enlargement of the predicted cavity with its volume, surface, and depth values.

Cavity studies associated with the mosquito sterol carrier protein are presented in Figure 2A, showing the biological receptor (orange) and the predicted cavity, characterized as cavity I (purple) [26]. This cavity predominantly appears in a specific region of the receptor, highlighting a potential site for interactions. Figure 2B presents a magnified view of the predicted cavity, emphasizing the most favorable region for complex formation. The cavity volume was 368.13 Å^3^, the surface area was 423.93 Å^2^, and the depth was 18.8 Å.

The amino acid residues forming the proposed cavity were identified, revealing that cavity I consists of 26 residues: Ala81, Glu103, Ile74, Ile106, Phe105, Gly75, Leu102, Leu109, Ser108, Met90, Met71, Ile99, Met66, Leu64, Phe32, Gln25, Arg24, Asn23, Val26, Arg15, Ile19, Asp20, Ile12, Leu16, Leu48, and Met46. These residues play a crucial role in forming the ligand-receptor complex within cavity I and may be associated with a network of interactions with reference residues.

The simulations for predicting possible receptor-associated cavities demonstrated a favorable environment for ligand–protein complex formation. The data in Figure 2 show the dominance of a relatively large cavity composed of 26 amino acid residues, suggesting a potentially favorable region for ligand interactions with the target receptor.

**Figure 3:**
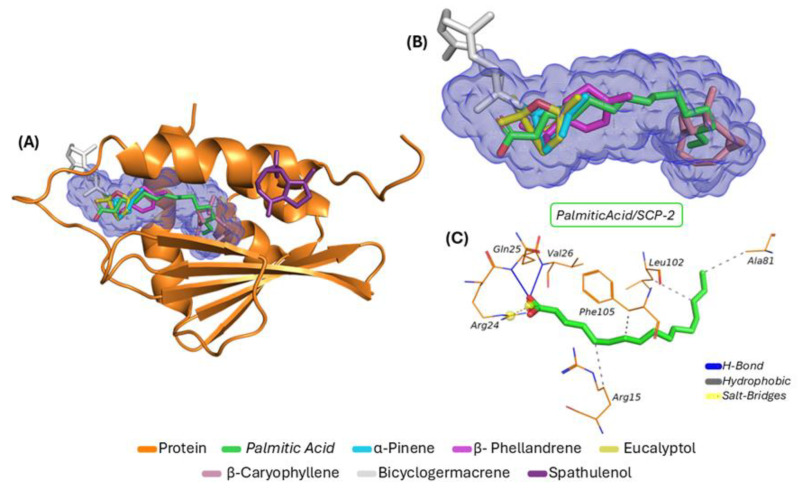
Complexes formed with the SCP-2 protein: (**A**) global view of the complexes within the predicted cavity; (**B**) magnified cavity view showing ligands with affinity for the pocket; and (**C**) interactions between the palmitic acid inhibitor and amino acid residues.

The molecular docking simulations presented in Figure 3A show all the complexes formed between the mosquito sterol carrier protein and the ligands, as follows: palmitic acid (green); *α-pinene* (cyan); *β-phellandrene* (pink); *eucalyptol* (yellow); *β-caryophyllene* (salmon); *bicyclogermacrene* (white), and *spathulenol* (purple). Their respective binding energy values were −6.2 kcal/mol, −6.0 kcal/mol, −6.8 kcal/mol, −6.0 kcal/mol, −5.9 kcal/mol, −4.7 kcal/mol, and −4.5 kcal/mol, while their RMSD values were 1.459 Å, 0.926 Å, 1.222 Å, 0.992 Å, 1.560 Å, 1.148 Å, and 1.867 Å, respectively. Figure 3B shows a magnified view of the predicted cavity containing all ligands that exhibited affinity for the binding pocket. The data indicate that the ligands (palmitic acid-inhibitor, *α-pinene*, *β-phellandrene*, *eucalyptol*, and *β-caryophyllene*) were fully inserted into the cavity, demonstrating high specificity compared to the co-crystallized inhibitor. Figure 3C highlights the interactions between the inhibitor and seven amino acid residues (Gln25, Val26, Arg24, Phe105, Leu102, Ala81, and Arg15), which were distributed among three interaction types: hydrogen bonds (H-bond), hydrophobic interactions, and salt bridges. The distances associated with each interaction type are shown in Table 3.

### 2.4. PAMPA Prediction

#### 2.4.1. Cell Effective Permeability Prediction

**Figure 4:**
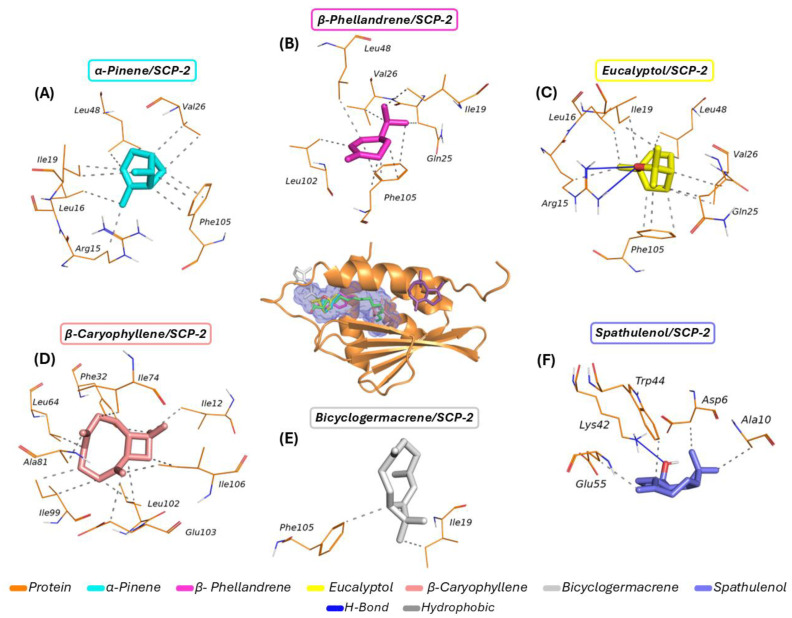
Molecular docking of the compounds with the SCP-2 receptor: (**A**) *α-pinene*/SCP-2 complex; (**B**) *β-phellandrene*/SCP-2 complex; (**C**) *eucalyptol*/SCP-2 complex; (**D**) *β-caryophyllene*/SCP-2 complex; (**E**) *bicyclogermacrene*/SCP-2 complex; and (**F**) *spathulenol*/SCP-2 complex.

According to the pharmacokinetic parameter classification system of Pfizer, Inc., (New York City, NY, USA) compounds with low lipophilicity at a physiological pH (logD < 2.0–4.0) exhibit characteristics indicative of effective cellular permeability, as evidenced by data obtained from PAMPA permeability assays. The observed *P*_app,A→B_ values in this context are typically in the range of ×10^−6^ cm/s in Caco-2 cell line models. Furthermore, this permeability profile is associated with high oral bioavailability due to the increased metabolic stability of the compounds [27,28]. MLP analyses revealed important characteristics regarding the polarity of the ligands (Figure 4).

#### 2.4.2. HLM Stability and Hepatic Clearance

The prediction of the site of metabolism is a crucial step in the estimation of metabolic activation. In the context of the oral absorption of small molecules by animals and humans, this issue is of particular interest. This process enables the reactivity of secondary metabolites to be predicted based on the chemical structure of the ligand [29,30]. The principle of the site of metabolism prediction is based on aligning structure–activity relationship (SAR) descriptors, which include structural sensitivity and specificity, and aims to identify biotransformation sites by testing similarity with known CYP450 fragments and substrates [29].

**Figure 5:**
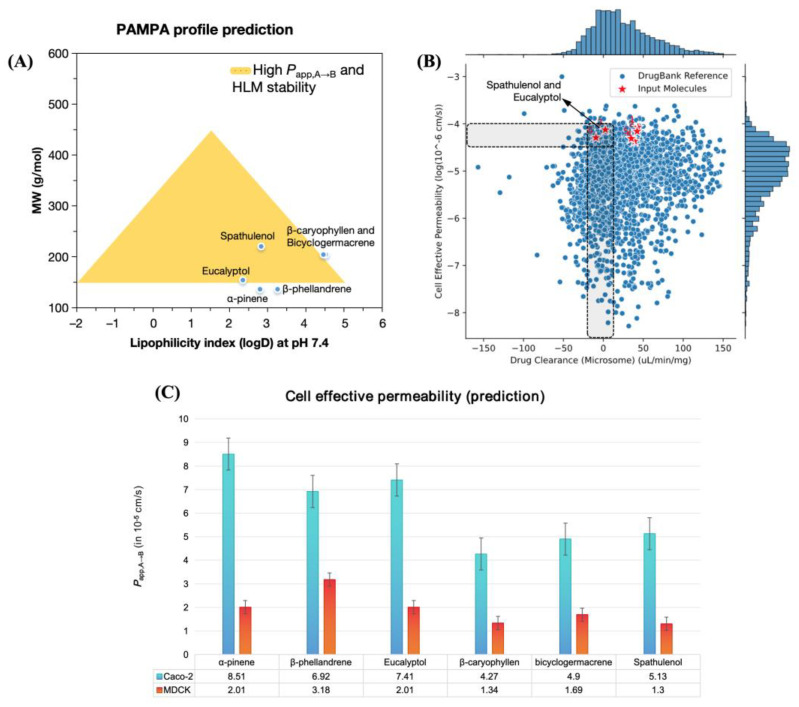
(**A**) alignment between MW and logD for estimation of the PAMPA profile for *P*_app,A→B_ and human liver microsome (HLM) stability; (**B**) similarity test with compounds deposited in the Drugbank^®^ database for the log*P*_app,A→B_ and *Cl*_Micro_ descriptors; and (**C**) prediction of cell effective permeability (PAMPA) in Caco-2 and MDCK cell lines.

Phase I metabolism, mediated by CYP450 isoenzymes (in cell hepatocytes), can result in the generation of reactive secondary metabolites, often formed through processes, such as epoxidation, which arise from the hydroxylation of aromatic centers [31,32]. These substructures may lead to metabolites capable of covalently binding to proteins and DNA, causing hepatic damage. Moreover, these biotransformation reactions can significantly impact hepatic clearance pathways, both in hepatic microsomes and hepatocytes, affecting oral bioavailability. The results of the site of metabolism prediction yielded highly interesting outcomes (Figure 5).

### 2.5. Ecotoxicity Risk Assessment

**Figure 6:**
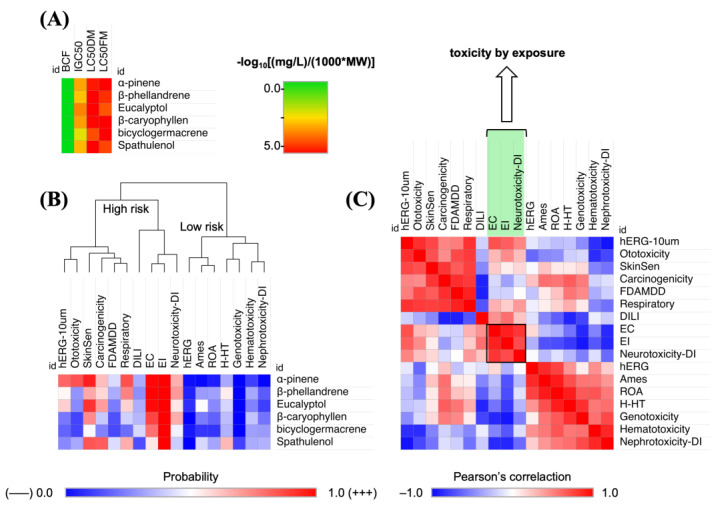
(**A**) Prediction of bioaccumulation factor (BCF) and acute toxicity by LC_50_ in *Fathead Minnow* (FM) and *Daphnia magna* (DM); (**B**) prediction of the probability of organic and exposure toxic response; and (**C**) Pearson’s correlation between toxicity models.

The prediction of ecotoxicity of chemical agents suspended in the air and water may imply the lethality of animal species underlying the affected ecosystem. The concentration capable of causing lethality in a population of fish in a specific time interval (96 h) can provide important information about the bioaccumulation factor of small organic compounds. The bioaccumulation factor is a measure that indicates the level at which these chemicals are distributed between the air and the aquatic environment [33]. As illustrated in Figure 6, the prediction results indicated that the compounds in question can induce an acute toxic response in aquatic species (logLC_50_ > 3.0 mg/L in Fathead Minnow) in addition to leading to exposure toxicity such as eye corrosion (EC) and eye irritation (EI).

## 3. Discussion

### 3.1. Chemical Composition

The chemical composition of *C. blanchetianus* essential oils revealed a predominance of monoterpenes and sesquiterpenes. Similar results were obtained in other studies on the same genus, which also demonstrated the prevalence of monoterpenes and sesquiterpenes [14,34,35]. Moreover, the major constituents of *C. blanchetianus* essential oils—*α-pinene*, *β-phellandrene*, *eucalyptol*, *β-caryophyllene*, *bicyclogermacrene*, and *spathulenol*—have been reported as main components in other *Croton* species. In some species, such as *C. rhamnifolioides*, *C. conduplicatus*, *C. decaryi*, and *C. geayi*, *β-caryophyllene* is the most abundant constituent [36,37].

The qualitative and quantitative variations observed in the chemical composition of the essential oils extracted at different times (8 a.m., 12 p.m., and 17 p.m.) may be attributed to environmental factors, including temperature, rainfall index, humidity, and soil type. The chemical composition of essential oils from aromatic plants is known to vary depending on environmental factors, as seen in *Ocimum gratissimum*, where the eugenol content ranged from 11% at 12 p.m. to 98% at 5 p.m. [38].

Seasonality also influences the chemical composition of essential oils. In *C. heliotropiifolius*, *β-caryophyllene* was identified as the main component, with levels varying from 46.99% in winter, 43.85% in spring, 41.04% in summer, and 28.61% in autumn [39]. Similarly, the chemical composition of *R. graveolens* leaf essential oil was strongly influenced by environmental factors, with its major compounds showing greater predominance during dry periods [40].

### 3.2. Larvicidal Activity

Essential oils are promising candidates for mosquito control as they are, in some cases, active against *Aedes aegypti*, readily available, and economically viable [23,41]. Recent studies have demonstrated the significant larvicidal activity of essential oils from *Croton* species. For instance, essential oils from the leaves, stalks, and inflorescences of *C. zehntneri* were tested at different concentrations against third-instar larvae of *Aedes aegypti*, showing LC_50_ values of 56.2, 51.3, and 57.5 μg/mL, respectively [19]. Similarly, essential oils from *C. jacobinensis* leaves, stalks, and inflorescences exhibited LC_50_ values of 79.3, 117.2, and 65.8 μg/mL [20].

The larvicidal activity of *C. blanchetianus* essential oils aligns with previous studies. Essential oils from aerial parts of *C. argyrophylloides* and *C. nepetaefolius* demonstrated LC_50_ values of 94.6 and 66.4 μg/mL, respectively [9], while those from *C. regelianus* leaves and stalks showed LC_50_ values of 84 and 24.22 μg/mL, respectively [42]. The different larvicidal activities observed can be attributed to variations in chemical composition.

The mortality percentages were calculated after 24 h; the larvicidal effects of these essential oils are shown in Table 2. The essential oils obtained at 8, 12, and 17 h showed LC_50_ values of 55.29 ± 3.21, 95.48 ± 2.68, and 64.88 ± 1.78 μg/mL, respectively. Therefore, the different activities of the essential oils can be attributed to the variations in their chemical compositions. Essential oils from the leaves of *C. blanchetianus* demonstrated larvicidal activity against *Aedes aegypti*, which can be explained by a possible relationship between larvicidal activity and the presence of monoterpenes (*α-pinene*, sabinene, β-pinene, myrcene, α-terpinene, and γ-terpinene) and sesquiterpenes (*β-caryophyllene* and *bicyclogermacrene*), which have been reported to be active against *Aedes aegypti* and can serve to increase the transmembrane absorption of lipophilic compounds, enhancing their larvicidal effects [23,24].

### 3.3. Molecular Docking

According to the molecular docking data, only *α-pinene*, *β-phellandrene*, *eucalyptol*, and *β-caryophyllene* exhibited entirely similar behavior to the co-crystallized inhibitor, indicating high specificity for the predicted binding pocket. In contrast, *bicyclogermacrene* was partially inserted into the interaction cavity, demonstrating lower specificity. Among all the evaluated ligands, only *spathulenol* showed affinity for a region entirely external to cavity I, indicating lower specificity for the primary biological target cavity.

The molecular docking simulations presented in Figure 4 illustrate the complexes formed and their respective interactions. Figure 4A shows *α-pinene* forming interactions with six amino acid residues (Leu48, Val26, Ile19, Leu16, Arg15, and Phe105). Figure 4B highlights *β-phellandrene*’s interactions with six residues (Leu48, Val26, Ile19, Gln25, Phe105, and Leu102). Figure 4C shows *eucalyptol* interacting with seven residues (Leu16, Ile19, Leu48, Val26, Gln25, Phe105, and Arg15). Figure 4D displays *β-caryophyllene* forming interactions with nine residues (Leu64, Phe32, Ile74, Ile12, Ala81, Ile99, Leu102, Ile106, and Glu103). Figure 4E reveals that *bicyclogermacrene* interacted with only two residues (Phe105 and Ile19), while Figure 4F shows *spathulenol* forming interactions with five residues (Trp44, Asp6, Ala10, Lys42, and Glu55). The interaction distances are presented in Table 3.

It is noteworthy that the high lipophilicity of the ligands promotes their binding to the lipid-binding site (e.g., palmitic acid) within the SCP-2 protein, particularly through the establishment of interactions with apolar side-chain residues, such as those derived from leucine (Leu), isoleucine (Ile), and valine (Val), in addition to aromatic residues derived from phenylalanine (Phe) [26,43].

The molecular docking results indicate that *α-pinene*, *β-phellandrene*, *eucalyptol*, and *β-caryophyllene* exhibited high specificity for the predicted binding site within cavity I, consistent with the palmitic acid, a fatty acid that is considered an endogenous substrate for SP2. Conversely, the bicyclogermacrene/SCP-2 complex was only partially inserted into the predicted cavity, interacting with some amino acid residues, demonstrating relatively similar interactions to the co-crystallized inhibitor. These findings suggest that these ligands exhibit a potentially favorable inhibitory effect on the target receptor. However, the spathulenol/SCP-2 complex did not form interactions similar to those observed with the palmitic acid, indicating a lower inhibitory potential for the mosquito sterol carrier protein.

### 3.4. PAMPA Prediction

#### 3.4.1. Molecular Lipophilicity Potential (MLP)

**Figure 7:**
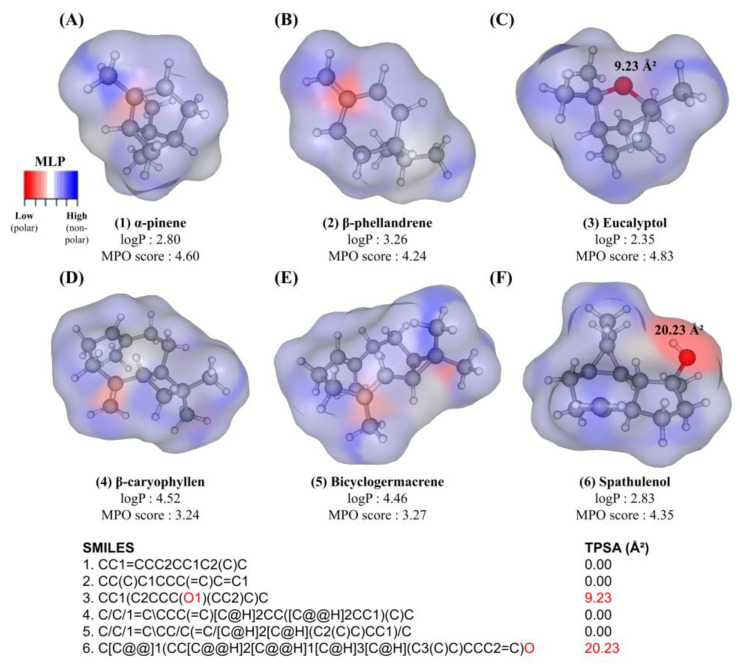
Molecular lipophilicity potential (MLP) surface of the compounds: (**A**) α-pinene; (**B**) β-phellandrene; (**C**) eucalyptol; (**D**) β-caryophyllen; (**E**) bicyclogermacrene; and (**F**) spathulenol. The color spectrum ranges from red (for polar fragments) to blue (for hydrophobic fragments). The figure also shows the prediction of the Topological Polar Surface Area (TPSA) with respect to molecular fragments from the Simplified Molecular Input Line Entry System (SMILES).

It was observed that natural products composed primarily of carbon and hydrogen exhibit a molecular surface predominantly accessible to hydrophobic environments (blue color spectra), with a strong contribution from terminal methyl groups (‘R-CH3) in the following compounds: (1) *α-pinene*, (2) *β-phellandrene*, (4) *β-caryophyllene*, and (5) *bicyclogermacrene*. However, the unsaturated centers improve aqueous solubility (red color spectra) (Figure 7), resulting in predicted logP values ranging from approximately 2.8 to 4.5 (Table 4).

On the other hand, compound (3) eucalyptol features a polar ‘R-O-R hydrogen bond acceptor group with a polar surface area of 9.23 Å^2^, resulting in a chemical species with a balance between lipophilicity and polarity (logD < 3) (Figure 7C). Meanwhile, the oxygenated hydrogen donor group ‘R-OH of the derivative (6) spathulenol exhibits a polar surface area of 20.23 Å^2^ (red color spectra), resulting in a lipophilicity index of approximately 2.83, indicating an optimal balance between the organic and aqueous phases (Figure 7F). These compounds showed MPO scores of 4.83 and 4.35, respectively, which fall within a range that indicates the formation of chemical species with proper alignment between lipophilicity, polarity (TPSA), and the PAMPA profile [44]. The results obtained provide insights into the contribution of polar groups to the cell permeability of the compounds.

#### 3.4.2. Cell Effective Permeability Prediction

By aligning the lipophilicity index at physiological pH (logD at pH 7.4) with MW, it was observed that the derivatives (3) *eucalyptol* and (6) *spathulenol* fit better into the ideal PAMPA profile estimated by Pfizer, Inc. (Figure 5A), particularly because they occupy a physicochemical space defined by MW 150–200 g/mol [27]. This empirical analysis is supported by the similarity test conducted with compounds deposited in the DrugBank^®^ database [45], where these compounds showed alignment with the physicochemical space occupied by compounds with low clearance in hepatic microsomes (ClMicro < 10 µL/min/kg) and high effective cellular permeability (log*P*_app,A→B_ > –4.5 cm/s), indicating that the absorption of these compounds results in high oral bioavailability (Figure 5B) [28].

When the predicted *P*_app,A→B_ values were analyzed for Caco-2 and MDCK cell lines, the cellular permeability of the compounds in more selective cells became evident. With predicted values on the order of 10^−5^ cm/s, it was observed that all compounds are permeable in more selective cell lines, with *P*_app,A→B_ MDCK > 1.0 × 10^−5^ cm/s (Figure 5C). Notably, compounds α-pinene, β-phellandrene, eucalyptol, and spathulenol presented predicted *P*_app,A→B_ Caco-2 values > 5.0 × 10^−5^ cm/s (Figure 5C), indicating that they are well-absorbed in the intestinal environment (Table 5) [46].

#### 3.4.3. HLM Stability and Hepatic Clearance

**Figure 8:**
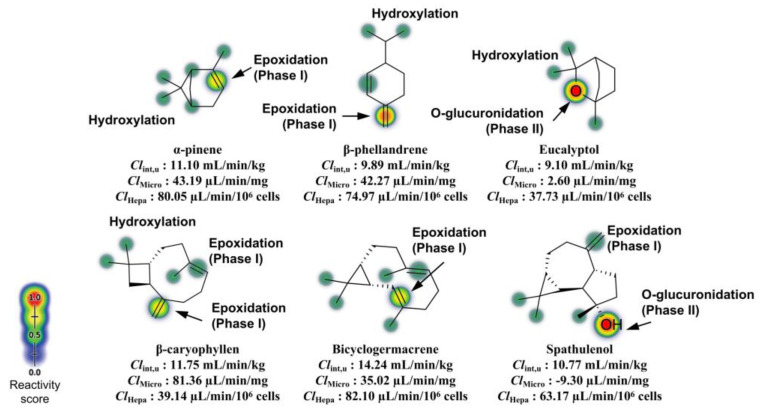
Prediction of the site of Phase I (CYP450-dependent) and Phase II (UGT-dependent) metabolism, and estimation of HLM stability with data expressed as intrinsic clearance (*Cl*_int,u_), clearance in hepatic microsomes (*Cl*_Micro_), and clearance in hepatocytes (*Cl*_Hepa_).

It was observed that the nonpolar derivatives β-caryophyllene and bicyclogermacrene have isolated alkenes as probable sites for epoxidation through aromatic hydroxylation, dependent on major CYP450 isoforms (2C9, 2D6, and 3A4) in the human liver (Figure 8), indicating the likelihood of organ toxicity through exposure. However, compound β-phellandrene contains a conjugated unsaturated system, increasing the probability of epoxidation at the terminal =CH2 double bond, showing the highest reactivity score for epoxidation (Figure 8).

On the other hand, the polar compounds eucalyptol and spathulenol exhibited oxygenated groups (‘R-O-R’ and ‘R-OH’, respectively) susceptible to O-glucuronidation dependent on UDP-glucuronosyltransferase (UGT) in Phase II (post-systemic) metabolism. This indicates that these compounds are less sensitive to Phase I hepatic metabolism and, therefore, exhibit higher metabolic stability.

This analysis is supported by hepatic clearance prediction, where the predicted ClMicro values of 2.60 µL/min/mg and −9.30 µL/min/mg for compounds eucalyptol and spathulenol, respectively, showed that these compounds may present better metabolic stability due to slow microsomal clearance (Table 6). Notably, the predicted value of −9.30 µL/min/mg for spathulenol reveals that this compound has structural similarity to compounds that tend to remain longer in the body [47], which may result in a negative difference between the rate of absorption and clearance. Additionally, compound eucalyptol stands out by showing the lowest clearance rate in hepatocytes, with a predicted ClHepa value of 37.73 µL/min/10^6^ cells, indicating that the compound’s polarity confers resistance to Phase I metabolism and slow clearance in hepatic cells.

### 3.5. PAMPA Prediction

The findings of the toxicity prediction in environmental biomarkers demonstrate that the lipophilicity of the compounds resulted in a low bioaccumulation factor (BCF), with an estimated volume of distribution of <2.0 L/kg for the polar compounds, i.e., the eucalyptol and spathulenol derivatives (see Table 6). These findings suggest a potential for secondary poisoning of the human organism through the food chain, demonstrating that the bioaccumulation potential of eucalyptol and spathulenol compounds in exposed biota is lower compared to that of other derivatives. However, the predicted LC_50_ values greater than 3.0 in the order of −log10(mg/L)/(1000 * MW) indicate that environmental exposure to these compounds (1–6) can cause acute lethality of 50% of a population of the aquatic species Fathead Minnow (FM) in 96 h, and 50% of a population of the planktonic crustacean Daphnia magna (DM) in 48 h (Figure 6A).

In predicting the probability of compounds inducing a toxic response, significant alerts of 1–6 were observed, resulting in eye corrosion (EC) and eye irritation (EI), in addition to neurotoxicity when absorbed by the human organism through the food chain. The EI descriptor was predicted with a probability >0.98; the EC descriptor was predicted with a probability between 0.5–0.98 (Figure 6B). Pearson’s correlation suggested a similarity of data between the EC, EI, and neurotoxicity descriptors, indicating that there is a greater toxic response by exposure than an organic toxic response (red color spectra in Figure 6C).

**Figure 9:**
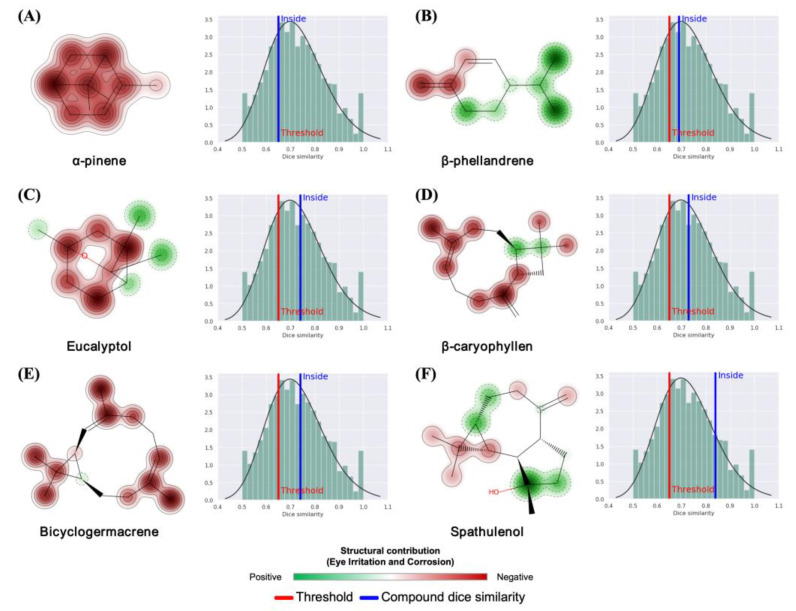
Prediction of structural contribution to the eye corrosion (EC) and eye irritation (EI) toxicity model for compounds: (**A**) α-pinene; (**B**) β-phellandrene; (**C**) eucalyptol; (**D**) β-caryophyllen; (**E**) bicyclogermacrene; and (**F**) spathulenol.

In the course of the analysis of the structural contributions to the toxicity endpoints, it was possible to observe that the isolated alkenes and tertiary carbons of the compounds contributed strongly to the EI and EC model (red color spectrum), as demonstrated in the 2D susceptibility map in Figure 9. From a statistical perspective, the molecular fragments are within the ideal dice similarity limits (>0.65), thereby ensuring a higher degree of confidence in the predictive tests (Figure 9). The results obtained demonstrate that, in terms of structure, these substances are derivatives capable of inducing irritability and certain contact toxic responses. This provides a more precise framework for predicting toxicity through exposure.

## 4. Materials and Methods

### 4.1. Plant Material

*C. blanchetianus* leaves were collected in Sobral County, Ceará State, Brazil (03°36′44″ S 40°18′37″ W). Plant authentication was performed by Professor Elantan Bezerra de Souza and a voucher specimen (#20433) was deposited at the Herbarium by professor Francisco José de Abreu Matos, State University Vale do Acaraú.

### 4.2. Extraction of the Essential Oils

Essential oils were extracted by hydrodistillation in a Clevenger-type graduated apparatus using a volumetric flask with a capacity of 2 L for 2 h [48]. The extractions were carried out on 1 kg samples of fresh leaves collected at 8:00 a.m., 12:00 p.m., and 17:00 p.m. (extraction was carried out on the same day as the material was collected). After being filtered and dried over anhydrous sodium sulfate, the isolated oils were stored in sealed glass vials, which were maintained under refrigeration at 4 °C until the GC–MS and GC–FID analyses. The essential oil yield was determined by dividing the total mass of oil obtained by 1 kg of fresh leaves [48]. The essential oil of leaves from *C. blanchetianus* appeared green. A yield of 0.02% of the essential oil was collected at 8:00 a.m.; 0.015% of the essential oil was collected at 12:00 p.m.; and 0.018% (*w*/*w*) of the essential oil was collected at 17:00 p.m.

### 4.3. Gas Chromatography–Flame Ionization Detection

GC–FID for the quantitative analysis was carried out on a Shimadzu (Kyoto, Japan) GC-17A gas chromatograph using a dimethylpolysiloxane DB-5 fused silica capillary column (30 mm × 0.25 mm, film thickness 0.25 m). H_2_ was used as the carrier gas at a flow rate of 1 mL/min and 30 psi inlet pressure; split, 1:30; a temperature program of 35–180 °C at 4 °C/min, then heated at a rate of 17 °C/min to 280 °C and kept isothermal for 10 min; the injector temperature was 250 °C; the detector used FID with a detector temperature of 250 °C [48].

### 4.4. Gas Chromatography–Mass Spectrometry

GC–MS for the analysis of the volatile constituents was carried out on a Hewlett-Packard Model 5971 GC–MS (American Laboratory Trading, East Lyme, CT, USA) using a non-polar DB-5 fused silica capillary column (30 mm × 0.25 mm i.d., 0.25 m film thickness); the carrier gas was helium, the flow rate was 1 mL/min with split ratio of 1:1. The injector temperature and detector temperature were 250 °C and 250 °C, respectively. The column temperature was programmed from 35 °C to 180 °C at 4 °C/min and then 180 °C to 250 °C at 10 °C/min. Mass spectra were recorded from 30–450 *m*/*z*. Individual components were identified by matching their 70 eV mass spectra with those of the spectrometer database using Wiley Online Library MS searches using retention indices as a preselection routine, as well as by visual comparison of the fragmentation pattern with those reported in the literature [48].

### 4.5. Larvicidal Bioassay

Essential oils were placed in beakers and dissolved in 20 mL H_2_O/DMSO 1.5% (*v*/*v*) at concentrations of 50–500 µg/mL, followed by the addition of 50 larvae at the third instar. For each experiment, both positive (Temephos at 3.22 µg/mL) and negative (distilled water containing 1.5% DMSO) control assays were carried out. Mortality was recorded after 24 h of exposure, during which no nutritional supplement was added. The experiments were carried out at 28 ± 2 °C. Each test was performed in triplicate. The bioassays were performed simultaneously under the same conditions for the essential oils from the leaves collected at 8 a.m., 12 p.m. and 17 p.m. Data were evaluated through regression analysis. From the regression line, the LC_50_ values were read representing the lethal concentration for 50% larval mortality of *Aedes aegypti*. The bioassays were performed at the Laboratório de Entomologia, Núcleo de Endemias, Secretaria de Saúde do Estado do Ceará, Brazil [49].

### 4.6. Statistical Analysis

The LC_50_ values of essential oil from leaves of *C. blanchetianus* were calculated using the probit analysis of the mortality data derived from bioassays [50].

### 4.7. Molecular Docking Simulations

Molecular docking simulations aim to elucidate the possible mechanisms of action of ligands through their interactions with biological targets, ranking chemical structures to propose potential drug candidates. The initial stages of the molecular docking simulations involved preparing the target receptor and candidate ligands to address *Aedes aegypti*. The standardization of the molecules *α-pinene*, *β-phellandrene*, *Eucalyptol*, *β-caryophyllene*, *Bicyclogermacrene*, and *Spathulenol* consisted of rendering the two-dimensional model using the academic license-supported software MarvinSketch^®^ version 24.1.0, Chemaxon© (https://chemaxon.com/marvin, accessed on 10 February 2025) [26]. For the preparation of the initial model, each molecule was individually input into the software Avogadro version 1.99.0 to convert the initial model into a three-dimensional structure. In this software, the MMFF94 (Merck Molecular Force Field) forcefield calculation was applied for each ligand [51,52]. The use of the forcefield aims to present structures with the lowest potential energy values, resulting in a more stable structure that is suitably adjusted for molecular docking simulations [53].

Theoretical studies on larvicidal activity against *Aedes aegypti* assessed the affinity behavior between drug-proposing ligands and the biological target through molecular docking simulation estimates, which allowed for the mechanism of action of each evaluated compound to be estimated. The crystalline structure of the mosquito’s sterol carrier protein (SCP-2) is stored in the virtual repository of the RCSB Protein Data Bank (https://www.rcsb.org/, accessed on 10 February 2025) under the identification code IdPDB: 1PZ4. This receptor is classified as a peptide-binding protein related to the *Aedes aegypti* organism, with its structure resolved by the X-ray diffraction method and a resolution of 1.35 Å [26,54].

To identify the possible cavities in the biological receptor, the *Proteinplus* virtual platform (Zentrum für Bioinformatik: Universität Hamburg—Proteins Plus Server) was used. The platform revealed all the amino acid residues forming the predicted cavity, where the presence of these residues provided an interaction pocket in the target receptor. The predicted region included defined values of volume (Å^3^), surface area (Å^2^), and depth (Å), showing available receptor sites for forming interactions between ligands and amino acid residues, ultimately forming a complex. The cavity study aimed to elucidate the possible amino acid residues in the pocket and propose a potential interaction route for the complex formation between the ligand and protein [55].

For the receptor standardization, it was necessary to remove residues (ligands and water molecules) present in the crystalline structure using Chimera software version 1.18 (build 42531) [56,57]. This step aimed to expose all possible protein cavities during molecular docking prediction studies. Additional refinements were carried out with the support of AutoDockTools™ by adding polar hydrogens and Gasteiger charges to the receptor [56,57]. For the biological receptor’s standardization, Grid-Box values were also adjusted to encompass the entire target receptor structure, with axis values set to x = 18.188, y = 28.577, z = 57.840, and dimensions of 87 Å × 75 Å × 81 Å (x, y, z), enabling the entire crystalline structure during the molecular docking simulations.

The molecular docking simulations were performed using AutoDockVina™ software version 1.2.5 [58,59], configured to carry out 50 independent simulations with 20 distinct results for each simulation between the ligands and the target receptor. This approach aimed to evaluate the possible interaction routes of the co-crystallized inhibitor palmitic acid (SCP-2 inhibitor) and to assess the potential interactions formed with the other simulated ligands. A comparative analysis strategy was thus outlined. The formation of the best poses was determined based on the convergence criterion of RMSD (Root Mean Square Deviation) equal to 2.0 Å [60].

### 4.8. Molecular Docking Simulations

For the investigation of passive diffusion in membranes, an in silico estimation of Parallel Artificial Membrane Permeability Assay (PAMPA) descriptors and metabolic stability was performed based on the chemical structure of the ligands [61]. The properties of PAMPA, expressed in terms of effective cell permeability (*P*_app,A→B_) in colorectal adenocarcinoma (Caco-2) and Madin–Darby canine kidney (MDCK) cell lines, were predicted to estimate the ability of the compounds to penetrate the larval cuticle of *Aedes aegypti* [46].

The two-dimensional pre-hydrogenized representations of the chemical structures were converted into Simplified Molecular Input Line Entry System (SMILES) linear notations using MarvinSketch^®^ software, version 24.1.0, Chemaxon© (https://chemaxon.com/marvin, accessed on 10 February 2025). This conversion was performed after conformational optimization using the Merk Molecular Force Field (MMFF94), in a very strict optimization limit, configured to return only the most stable conformer with the lowest energy, where the molecular lipophilicity potential (MLP) was estimated—Equation (1)—along with the multiparameter optimization (MPO) score—Equation (2), as follows:

(1) $${MLP}_{k}=\sum_{i=1}^{N}f_{i}\cdot F\left(d_{ij}\right)$$

(2) $$D=\sum_{k=1}^{N}w_{k}T_{k}\left(x_{k}^{0}\right)$$

The MLP estimation function—Equation (1)—takes into account the lipophilicity coefficient (*f_i_*) of each molecular fragment (*i*), assigning a positive value to lipophilic fragments and a negative value to hydrophilic or polar fragments. This is contingent upon a total of *N* fragments for each molecule. The *F*(*d_ij_*) factor is a function of the spatial distance between each pair of fragments. It is applied to measure structural similarity based on the superposition of two structures, *i* and *j*. This is also termed the Fermi distance function [62]. The results manifest as a surface map where the color spectrum ranges from blue (for lipophilic fragments) to red (for polar fragments).

These results were compared to the descriptors of lipophilicity by the partition coefficient (logP) and polarity, estimated by the Topological Polar Surface Area (TPSA), resultante do somatório das áreas de superfície polar (PSA)—in Å^2^—dos grupos R-OH (20.23 Å^2^) doadores de H-bond (HBD) e R_2_O (9.23 Å^2^) aceitadores de H-bond (HBA), conforme o modelo de cálculo de TPSA estabelecido por Ertl (2007) [63].

The MPO is a statistical function utilized here to evaluate the degree of desirability (*D*)—Equation (2)—of each physicochemical property *k* associated with cell permeability and clearance. The selection of smaller and more polar compounds was accompanied by the assignment of a weight factor (*w*) that varied from 0, for properties with x-values calculated outside the ideal statistical threshold (*x*_a_ < *x*_k_), to 1, for x-values calculated within the ideal statistical spectrum (*x*_k_ ≤ *x*_b_). The desirability functions consider unilateral limits, formed by logP ≤ 3, logD ≤ 2, molecular weight (MW) ≤ 360 g/mol, H-bond donors (HBD) ≤ 1 and pKa ≤ 8, and the bilateral function of 40 < TPSA ≤ 90 Å^2^, since low-polar compounds can be toxic and high-polar compounds can have their pharmacokinetics reduced. The sum of the *w*-factors, associated with the desirability thresholds (*T*(*x*)), results in a score ranging from 0, for compounds with unfeasible pharmacokinetics, to 6, for compounds where all six physicochemical properties have been met (*N* = 6 properties) [64]. The selection of physicochemical descriptors is oriented towards the analysis of the lipophilicity profile (logP and logD), size and polarity (MW and TPSA) and acidity/basicity balance (HBD and pKa). These factors exert a significant influence on the absorption, metabolic stability, and oral bioavailability of drugs.

Subsequently, the ADMETlab 3.0 (https://admetlab3.scbdd.com/, accessed on 10 February 2025) and ADMET-AI (https://admet.ai.greenstonebio.com/, accessed on 10 February 2025) servers were utilized to estimate the pharmacokinetic properties, derived from in vitro assays, from the SMILES notation generated for the compounds. The descriptors are expressed in PAMPA properties, which include, as follows: *P*_app,A→B_ in Caco-2 and MDCK cell lines; clearance in human liver microcosmic system (ClMicro) and hepatocytes (ClHepa); and systemic distribution descriptors, expressed in P-glycoprotein (P-gp) substrate, volume of distribution at steady state (Vdss), and Plasma Protein Binding (PPB). The results obtained were related to the metabolic stability test, which was performed by predicting the metabolism site using the XenoSite server (https://xenosite.org/, accessed on 10 February 2025). This resulted in a sensitivity map of the compounds being metabolized by metabolic isoenzymes of cytochrome P450 (CYP450) [65].

### 4.9. Ecotoxicity Prediction

The SMILES code of the compounds was subjected to a systematic prediction of ecotoxicity, considering organic and exposure toxicity endpoints, using the ADMETlab 3.0 tool (https://admetlab3.scbdd.com/, accessed on 10 February 2025). The endpoints encompass, as follows: the bioaccumulation factor (BCF); growth inhibition (IGC_50_) in aquatic protozoa of the species Tetrahymena pyriformis; the lethal concentration (LC_50_) in fathead minnow (96 h) and daphnia magna (48 h); organ toxicity based on cardiotoxicity, ototoxicity, skin sensitivity, carcinogenicity, Ames mutagenicity, rat acute toxicity (ROA), human hepatotoxicity (H-HT), hematotoxicity, nephrotoxicity; and exposure toxicity based on eye corrosion (EC), eye irritation (EI), and neurotoxicity. The estimated probability values were then analyzed using the Morpheus statistical tool (https://software.broadinstitute.org/morpheus/, accessed on 10 February 2025). This analysis resulted in graphical inspections based on a heatmap.

### 4.10. Statistical Analysis

The LC_50_ value of essential oil from leaves of *C. blanchetianus* was calculated using the probit analysis of the mortality data derived from bioassays [25].

## 5. Conclusions

The circadian study results emphasize the importance of collection time on the chemical composition of essential oils from *C. blanchetianus*, which directly influences their larvicidal activity against *Aedes aegypti*. The variation in LC_50_ values throughout the day suggests that the effectiveness of bioactive compounds can be optimized by adjusting the extraction timing. The strong interaction of α-pinene, β-phellandrene, eucalyptol, and β-caryophyllene with the active site of the sterol transport protein highlights their role in larvicidal activity, reinforcing the potential of essential oils for sustainable vector control. Additionally, predictive cell permeability tests showed a PAMPA profile indicating high effective cell permeability and reduced clearance in microsomes and hepatocytes. This suggests that eucalyptol and spathulenol have increased permeability and minimal secretion from cells, characterizing them as compounds with enhanced cell viability. Thus, *C. blanchetianus* essential oils offer a safe alternative for combating dengue.

## Figures and Tables

**Table 1:** Chemical composition of the essential oils from the leaves of *C. blanchetianus* obtained at different plant material collection times.

Compounds	IK *_literature_	IK_experimental_		Relative Percentage	
			8 h	12 h	17 h
α-Thujene	930	924	0.78	3.01	1.07
α-Pinene	939	932	6.17	10.56	7.07
Sabinene	975	969	0.92	1.97	1.49
β-Pinene	979	974	0.89	1.18	
Myrcene	990	988	5.11	3.96	6.32
α-Phellandrene	1002	1002	1.22	2.02	1.55
δ-3-Carene	1011	1008		0.56	
α-Terpinene	1017	1014		0.66	
*O*-Cymene	1026	1022	1.18	2.25	1.21
β-Phellandrene	1029	1025	9.55	10.20	13.26
Eucalyptol	1031	1026	11.45	18.99	14.68
γ-Terpinene	1059	1053	0.74	1.13	1.35
Terpinolene	1088	1086	1.47	0.94	
Linalool	1096	1095	0.73	0.97	
Terpinen-4-ol	1177	1173		1.37	
α-Terpineol	1188	1186	1.28	1.58	
β-Elemene	1390	1389		0.66	
Sativene	1391	1390	2.37	4.06	4.67
β-caryophyllene	1419	1417	9.91	7.90	9.42
Aromadendrene	1441	1439	0.98		
6,9-Guaiadiene	1444	1442	1.38		4.95
α-Caryophyllene	1454	1452	1.45	1.23	
γ-Muurolene	1479	1478	2.79	2.85	2.51
Bicyclogermacrene	1500	1500	10.74	8.59	11.32
δ-Cadinene	1523	1522	1.01	0.66	
Spathulenol	1578	1577	12.85	7.91	12.49
Caryophyllene oxide	1583	1582	4.59	3.06	6.64
Total			89.56	98.27	100.00

* IK—Kovats index.

**Table 2:** LC_50_ values of *C. blanchetianus* essential oils.

Essential Oils	LC_50_ (μg/mL)
Essential oil (8 h)	55.29 ± 3.21
Essential oil (12 h)	95.48 ± 2.68
Essential oil (17 h)	64.88 ± 1.78
Temephos^®^	1.40 ± 0.20

**Table 3:** Types of interactions formed between ligands and the SCP-2 protein.

Ligand	Interaction Type	Residue (Distance in Å)
Palmitic acid	Hydrophobic	Arg15 (3.97), Ala81 (3.98), Leu102 (3.96), Phe105 (3.53)
	H-Bond	Gln25 (1.69), Val26 (2.03)
	Salt Bridges	Arg24 (3.92)
α-pinene	Hydrophobic	Arg15 (3.52), Leu16 (3.75), Ile19 (3.27), Ile19 (3.78), Val26 (3.71), Val26 (3.68), Leu48 (3.43), Leu48 (3.62), Phe105 (3.63), Phe105 (3.55), Phe105 (3.50)
β-phellandrene	Hydrophobic	Ile19 (3.98), Gln25 (3.53), Val26 (3.77), Leu48 (3.68), Leu102 (3.79), Phe105 (3.71), Phe105 (3.60), Phe105 (3.88), Phe105 (3.55), Phe105 (3.95)
Eucalyptol	Hydrophobic	Arg15 (3.71), Leu16 (3.77), Ile19 (3.88) Ile19 (3.43), Gln25 (3.72), Val26 (3.66), Val26 (3.98), Leu48 (3.40), Leu48 (3.68), Phe105 (3.59), Phe105 (3.52), Phe105 (3.19)
	H-Bond	Arg15 (3.17), Arg15 (3.21)
β-caryophyllene	Hydrophobic	Ile12 (3.72), Phe32 (3.32), Phe32 (3.81), Leu64 (2.88), Ile74 (3.61), Ala81 (3.06) Ile99 (3.34), Ile99 (3.91), Leu102 (3.70), Leu102 (3.53), Glu103 (3.71), Ile106 (3.54), Ile106 (2.96)
Bicyclogermacrene	Hydrophobic	Ile19 (3.52), Phe105 (3.60)
Spathulenol	Hydrophobic	Asp6 (3.59), Ala10 (3.86), Trp44 (3.69), Glu55 (3.65)
	H-Bond	Lys42 2.46

Legend: Ala (Alanine), Arg (Arginine), Asp (Aspartate), Gln (Glutamine), Glu (Glutamate), Ile (Isoleucine), Leu (Leucine), Lys (Lysine), Phe (Phenylalanine), Val (Valine), Trp (Tryptophan).

**Table 4:** Physicochemical properties calculated and applied to the Pfizer classification system using the MPO algorithm.

Compound	logP	logD	MW (g/mol)	TPSA (Å^2^)	HBD	pKa	MPO Score
α-pinene	2.80	2.80	136.24	0.00	0	NIA	4.60
β-phellandrene	3.26	3.26	136.24	0.00	0	NIA	4.24
Eucalyptol	2.35	2.35	154.25	9.23	0	−4.21 ‘R-O-R	4.83
β-caryophyllen	4.52	4.52	204.36	0.00	0	NIA	3.24
Bicyclogermacrene	4.46	4.46	204.36	0.00	0	NIA	3.27
Spathulenol	2.83	2.83	220.36	20.23	1	−0.64 ‘R-OH	4.35

Legend: logD (distribution coefficient); MW (molecular weight), TPSA (topological polar surface area), HBD (H-bond donors), MPO (multiparameter optimization).

**Table 5:** Pharmacokinetic properties of effective cell permeability expressed in *P*_app,A→B_ Caco-2 and MDCK, passive efflux by P-gp and PAMPA profile.

Compound	*P*_app,A→B_ (Cell Permeability)		P-gp Efflux	PAMPA Class
	Caco-2 (cm/s)	MDCK (cm/s)		
α-pinene	8.51 × 10^−5^	2.01 × 10^−5^	0.01	High
β-phellandrene	6.92 × 10^−5^	3.18 × 10^−5^	0.02	High
Eucalyptol	7.41 × 10^−5^	2.01 × 10^−5^	0.01	High
β-caryophyllen	4.27 × 10^−5^	1.34 × 10^−5^	0.08	High
Bicyclogermacrene	4.90 × 10^−5^	1.69 × 10^−5^	0.07	High
Spathulenol	5.13 × 10^−5^	1.30 × 10^−5^	0.10	High

Legend: *P*_app,A→B_ (cell effective permeability), Caco-2 (adenocarcinoma colorectal), Madin–Darby Canine Kidney (MDCK), P-gp (P-glycoprotein substrate), PAMPA (Parallel Artificial Membrane Permeability Assay).

**Table 6:** Stability properties in the human liver microsome (HLM) expressed in descriptors of hepatic clearance and systemic distribution.

Compound	HLM Stability			BCF (L/kg)
	*Cl*_int,u_ (mL/min/kg)	*Cl*_Micro_ (µL/min/mg)	*Cl*_Hepa_ (mL/min/10^6^ cells)	
α-pinene	11.10	43.19	80.05	2.93
β-phellandrene	9.89	42.27	74.97	2.47
Eucalyptol	9.10	2.60	37.73	0.27
β-caryophyllen	11.75	81.36	39.14	2.86
Bicyclogermacrene	14.24	35.02	82.10	3.58
Spathulenol	10.77	-9.30	63.17	1.78

Legend: bioaccumulation factor (BCF), intrinsic cellular clearance (*Cl*_int,u_), clearance in liver microsomes (*Cl*_Micro_) and clearance in hepatocytes (*Cl*_Hepa_).

## Data Availability

Data are contained within the article.
